# Supplementary material for: Meta-Analysis and Gene Set Enrichment Relative to ER Status Reveal Elevated Activity of MYC and E2F in the “Basal” Breast Cancer Subgroup
Source: PLoS One. 2009 Mar 9;4(3):e4710. doi: 10.1371/journal.pone.0004710 (PMC2650420; doi:10.1371/journal.pone.0004710)
Supplement: Dataset S1 — GSEA results from first screen. The file contains a zipped archive of the HTML results of the second GSEA screen. Reports for enriched or depleted datasets may be accessed from the “index.html” link within the my_analysis.GseaPreranked.1219975950625.rpt.zip ZIP archive. Enrichment plots are available for each of the 50 most enriched and depleted gene sets. (13.32 MB ZIP) [file pone.0004710.s001.zip › my_analysis.GseaPreranked.1219975950625.rpt.zip!//HCC_SURVIVAL_GOOD_VS_POOR_DN.html]

Details for gene set HCC\_SURVIVAL\_GOOD\_VS\_POOR\_DN[GSEA]

|  || Dataset | zSco.ERneg.to.ERpos\_collapsed |
| Phenotype | NoPhenotypeAvailable |
| Upregulated in class | na\_pos |
| GeneSet | HCC\_SURVIVAL\_GOOD\_VS\_POOR\_DN |
| Enrichment Score (ES) | 0.56671476 |
| Normalized Enrichment Score (NES) | 2.3958118 |
| Nominal p-value | 0.0 |
| FDR q-value | 0.0 |
| FWER p-Value | 0.0 |
Table: GSEA Results Summary

  

Fig 1: Enrichment plot: HCC\_SURVIVAL\_GOOD\_VS\_POOR\_DN      
 Profile of the Running ES Score & Positions of GeneSet Members on the Rank Ordered List

  

| PROBE | GENE SYMBOL | GENE\_TITLE | RANK IN GENE LIST | RANK METRIC SCORE | RUNNING ES | CORE ENRICHMENT || 1 | TES |  |  | 8 | 7.200 | 0.0226 | Yes |
| 2 | TTK |  |  | 11 | 7.059 | 0.0453 | Yes |
| 3 | HDAC2 |  |  | 32 | 6.511 | 0.0648 | Yes |
| 4 | CCNA2 |  |  | 52 | 6.297 | 0.0836 | Yes |
| 5 | KLF5 |  |  | 87 | 5.966 | 0.1003 | Yes |
| 6 | ODC1 |  |  | 94 | 5.931 | 0.1190 | Yes |
| 7 | SNRPG |  |  | 142 | 5.628 | 0.1336 | Yes |
| 8 | PELI1 |  |  | 162 | 5.500 | 0.1499 | Yes |
| 9 | DEK |  |  | 190 | 5.364 | 0.1652 | Yes |
| 10 | MCM6 |  |  | 198 | 5.317 | 0.1818 | Yes |
| 11 | MTHFD2 |  |  | 205 | 5.307 | 0.1985 | Yes |
| 12 | RPS7 |  |  | 216 | 5.243 | 0.2146 | Yes |
| 13 | USP1 |  |  | 222 | 5.212 | 0.2311 | Yes |
| 14 | CKS2 |  |  | 239 | 5.145 | 0.2465 | Yes |
| 15 | CENPF |  |  | 268 | 5.028 | 0.2606 | Yes |
| 16 | PDCD5 |  |  | 270 | 5.025 | 0.2767 | Yes |
| 17 | MCM7 |  |  | 289 | 4.970 | 0.2914 | Yes |
| 18 | CSDA |  |  | 337 | 4.835 | 0.3034 | Yes |
| 19 | ATP5C1 |  |  | 471 | 4.463 | 0.3078 | Yes |
| 20 | NOL5A |  |  | 502 | 4.345 | 0.3195 | Yes |
| 21 | DLG7 |  |  | 521 | 4.306 | 0.3320 | Yes |
| 22 | CCT6A |  |  | 566 | 4.210 | 0.3423 | Yes |
| 23 | NFE2L3 |  |  | 570 | 4.204 | 0.3556 | Yes |
| 24 | MARCKS |  |  | 613 | 4.107 | 0.3657 | Yes |
| 25 | MCM2 |  |  | 630 | 4.080 | 0.3776 | Yes |
| 26 | ACTR3 |  |  | 724 | 3.892 | 0.3832 | Yes |
| 27 | HIST1H4C |  |  | 733 | 3.875 | 0.3951 | Yes |
| 28 | HRB |  |  | 752 | 3.840 | 0.4061 | Yes |
| 29 | YWHAQ |  |  | 774 | 3.803 | 0.4168 | Yes |
| 30 | ROD1 |  |  | 855 | 3.700 | 0.4227 | Yes |
| 31 | S100A6 |  |  | 880 | 3.653 | 0.4326 | Yes |
| 32 | UBE2D1 |  |  | 931 | 3.589 | 0.4404 | Yes |
| 33 | UBA2 |  |  | 950 | 3.552 | 0.4505 | Yes |
| 34 | DSG2 |  |  | 1068 | 3.393 | 0.4526 | Yes |
| 35 | RGS2 |  |  | 1093 | 3.369 | 0.4617 | Yes |
| 36 | FBL |  |  | 1116 | 3.344 | 0.4708 | Yes |
| 37 | ANXA3 |  |  | 1142 | 3.317 | 0.4796 | Yes |
| 38 | NP |  |  | 1241 | 3.199 | 0.4825 | Yes |
| 39 | MSH6 |  |  | 1276 | 3.164 | 0.4901 | Yes |
| 40 | SET |  |  | 1290 | 3.148 | 0.4993 | Yes |
| 41 | PCNA |  |  | 1346 | 3.087 | 0.5051 | Yes |
| 42 | HIF1A |  |  | 1396 | 3.044 | 0.5112 | Yes |
| 43 | RPS6 |  |  | 1557 | 2.898 | 0.5084 | Yes |
| 44 | NCBP2 |  |  | 1601 | 2.850 | 0.5144 | Yes |
| 45 | CCNB1 |  |  | 1626 | 2.823 | 0.5217 | Yes |
| 46 | RPS18 |  |  | 1682 | 2.771 | 0.5264 | Yes |
| 47 | RPE |  |  | 1698 | 2.762 | 0.5342 | Yes |
| 48 | SERPINH1 |  |  | 1861 | 2.639 | 0.5305 | Yes |
| 49 | MRPL42 |  |  | 1901 | 2.605 | 0.5359 | Yes |
| 50 | RALA |  |  | 2015 | 2.517 | 0.5355 | Yes |
| 51 | ECT2 |  |  | 2026 | 2.509 | 0.5428 | Yes |
| 52 | RAN |  |  | 2205 | 2.385 | 0.5370 | Yes |
| 53 | VCL |  |  | 2276 | 2.334 | 0.5393 | Yes |
| 54 | ITPR3 |  |  | 2329 | 2.308 | 0.5428 | Yes |
| 55 | BCAT1 |  |  | 2344 | 2.290 | 0.5491 | Yes |
| 56 | CCT5 |  |  | 2398 | 2.262 | 0.5524 | Yes |
| 57 | CDK4 |  |  | 2546 | 2.176 | 0.5483 | Yes |
| 58 | ANXA4 |  |  | 2572 | 2.160 | 0.5533 | Yes |
| 59 | RPL31 |  |  | 2638 | 2.125 | 0.5553 | Yes |
| 60 | PTMA |  |  | 2647 | 2.119 | 0.5615 | Yes |
| 61 | LXN |  |  | 2669 | 2.106 | 0.5667 | Yes |
| 62 | SLBP |  |  | 2921 | 1.974 | 0.5541 | No |
| 63 | RPL38 |  |  | 3061 | 1.903 | 0.5497 | No |
| 64 | LSM8 |  |  | 3317 | 1.787 | 0.5361 | No |
| 65 | NAP1L1 |  |  | 3421 | 1.740 | 0.5339 | No |
| 66 | EEF1E1 |  |  | 3449 | 1.730 | 0.5375 | No |
| 67 | TOPBP1 |  |  | 3475 | 1.722 | 0.5411 | No |
| 68 | KNTC1 |  |  | 3562 | 1.679 | 0.5400 | No |
| 69 | PFDN4 |  |  | 3633 | 1.647 | 0.5401 | No |
| 70 | RWDD1 |  |  | 3707 | 1.619 | 0.5397 | No |
| 71 | HMGB2 |  |  | 3883 | 1.558 | 0.5315 | No |
| 72 | NPM1 |  |  | 4288 | 1.397 | 0.5054 | No |
| 73 | K-ALPHA-1 |  |  | 4312 | 1.387 | 0.5082 | No |
| 74 | NALP2 |  |  | 4369 | 1.367 | 0.5083 | No |
| 75 | RPL12 |  |  | 4551 | 1.302 | 0.4988 | No |
| 76 | SNRPB2 |  |  | 4602 | 1.286 | 0.4992 | No |
| 77 | KIAA0101 |  |  | 4770 | 1.230 | 0.4905 | No |
| 78 | RPL35 |  |  | 4951 | 1.172 | 0.4806 | No |
| 79 | HN1 |  |  | 4953 | 1.171 | 0.4843 | No |
| 80 | RBX1 |  |  | 4966 | 1.166 | 0.4872 | No |
| 81 | HIST1H4A |  |  | 5241 | 1.067 | 0.4699 | No |
| 82 | MAPRE1 |  |  | 5601 | 0.944 | 0.4457 | No |
| 83 | KIF5B |  |  | 5668 | 0.924 | 0.4437 | No |
| 84 | OLIG2 |  |  | 5815 | 0.873 | 0.4355 | No |
| 85 | KHDRBS1 |  |  | 5819 | 0.872 | 0.4381 | No |
| 86 | RPS3 |  |  | 5994 | 0.814 | 0.4275 | No |
| 87 | DYRK2 |  |  | 6103 | 0.782 | 0.4218 | No |
| 88 | SLC34A1 |  |  | 6142 | 0.771 | 0.4215 | No |
| 89 | RAB32 |  |  | 6275 | 0.724 | 0.4138 | No |
| 90 | RPL27 |  |  | 6815 | 0.546 | 0.3747 | No |
| 91 | CTBP2 |  |  | 7675 | 0.239 | 0.3104 | No |
| 92 | CLIC1 |  |  | 8141 | 0.060 | 0.2754 | No |
| 93 | DDX48 |  |  | 8171 | 0.050 | 0.2733 | No |
| 94 | RPS9 |  |  | 8334 | -0.008 | 0.2611 | No |
| 95 | YWHAH |  |  | 8629 | -0.142 | 0.2393 | No |
| 96 | BZW2 |  |  | 8676 | -0.160 | 0.2363 | No |
| 97 | MYH2 |  |  | 8698 | -0.167 | 0.2353 | No |
| 98 | MMD |  |  | 8749 | -0.188 | 0.2321 | No |
| 99 | LAMB1 |  |  | 9080 | -0.350 | 0.2082 | No |
| 100 | ATP2B1 |  |  | 9376 | -0.495 | 0.1875 | No |
| 101 | BUB3 |  |  | 9557 | -0.594 | 0.1757 | No |
| 102 | RPS3A |  |  | 9568 | -0.601 | 0.1769 | No |
| 103 | CALM2 |  |  | 9592 | -0.613 | 0.1772 | No |
| 104 | HNRPC |  |  | 9598 | -0.616 | 0.1788 | No |
| 105 | RPL9 |  |  | 9637 | -0.636 | 0.1779 | No |
| 106 | RPS5 |  |  | 9643 | -0.640 | 0.1796 | No |
| 107 | NTS |  |  | 9685 | -0.661 | 0.1786 | No |
| 108 | HMGN1 |  |  | 9754 | -0.697 | 0.1757 | No |
| 109 | PLP2 |  |  | 9852 | -0.755 | 0.1708 | No |
| 110 | OPLAH |  |  | 9896 | -0.780 | 0.1701 | No |
| 111 | CD164 |  |  | 10182 | -0.944 | 0.1515 | No |
| 112 | RPL17 |  |  | 10246 | -0.985 | 0.1500 | No |
| 113 | HNRPA1 |  |  | 10609 | -1.250 | 0.1266 | No |
| 114 | TETRAN |  |  | 11410 | -1.943 | 0.0722 | No |
| 115 | KRT10 |  |  | 11580 | -2.108 | 0.0662 | No |
| 116 | CD2AP |  |  | 11908 | -2.444 | 0.0493 | No |
| 117 | PBX1 |  |  | 12018 | -2.582 | 0.0494 | No |
| 118 | IER3 |  |  | 12423 | -3.140 | 0.0289 | No |
| 119 | YWHAB |  |  | 12693 | -3.604 | 0.0202 | No |
| 120 | NSF |  |  | 12697 | -3.607 | 0.0316 | No |
| 121 | KRT19 |  |  | 13097 | -4.808 | 0.0169 | No |
Table: GSEA details [plain text format]

  

Fig 2: HCC\_SURVIVAL\_GOOD\_VS\_POOR\_DN: Random ES distribution      
 Gene set null distribution of ES for **HCC\_SURVIVAL\_GOOD\_VS\_POOR\_DN**
